# Gene expression profiling in necrotizing enterocolitis reveals pathways common to those reported in Crohn’s disease

**DOI:** 10.1186/s12920-016-0166-9

**Published:** 2016-01-22

**Authors:** Éric Tremblay, Marie-Pier Thibault, Emanuela Ferretti, Corentin Babakissa, Valérie Bertelle, Marcos Bettolli, Karolina Maria Burghardt, Jean-François Colombani, David Grynspan, Emile Levy, Peng Lu, Sandeep Mayer, Daniel Ménard, Olivier Mouterde, Ingrid B. Renes, Ernest G. Seidman, Jean-François Beaulieu

**Affiliations:** 1 Department of Anatomy and Cell Biology, Faculté de Médecine et Sciences de la Santé, Université de Sherbrooke, 3001, 12th Avec North, J1H 5N4 Sherbrooke, QC Canada; 2 Division of Neonatology, Department of Pediatrics, CHEO, Ottawa, ON Canada; 3 Department of Pediatrics, Faculté de Médecine et Sciences de la Santé, Université de Sherbrooke, Sherbrooke, QC Canada; 4 Division of Neonatology, Department of Pediatrics, Faculté de Médecine et Sciences de la Santé, Université de Sherbrooke, Sherbrooke, QC Canada; 5 Department of Surgery, CHEO, Ottawa, ON Canada; 6 Division of Gastroenterology, Hepatology & Nutrition, CHEO, Ottawa, ON Canada; 7 Department of Pediatrics, CHU de Martinique, Fort-de-France, France; 8 Department of Pathology and Laboratory Medicine, Faculty of Medicine, University of Ottawa, Ottawa, ON Canada; 9 Department of Nutrition, Centre de recherche, CHU Sainte-Justine, Université de Montréal, Montréal, QC Canada; 10 Department of Pediatrics, Erasmus MC-Sophia, Rotterdam, The Netherland; 11 Department of Surgery, Faculté de Médecine et Sciences de la Santé, Université de Sherbrooke, Sherbrooke, QC Canada; 12 CHU de Rouen, Department of Medical Pediatrics, Rouen, France; 13 Emma Children’s Hospital-AMC, Amsterdam, The Netherlands; 14 Division of Gastroenterology, McGill University, Montréal, QC Canada

**Keywords:** Human intestine, Preterm birth, Transcriptomics, Gene expression, Immune response

## Abstract

**Background:**

Necrotizing enterocolitis (NEC) is the most frequent life-threatening gastrointestinal disease experienced by premature infants in neonatal intensive care units. The challenge for neonatologists is to detect early clinical manifestations of NEC. One strategy would be to identify specific markers that could be used as early diagnostic tools to identify preterm infants most at risk of developing NEC or in the event of a diagnostic dilemma of suspected disease. As a first step in this direction, we sought to determine the specific gene expression profile of NEC.

**Methods:**

Deep sequencing (RNA-Seq) was used to establish the gene expression profiles in ileal samples obtained from preterm infants diagnosed with NEC and non-NEC conditions. Data were analyzed with Ingenuity Pathway Analysis and ToppCluster softwares.

**Results:**

Data analysis indicated that the most significant functional pathways over-represented in NEC neonates were associated with immune functions, such as altered T and B cell signaling, B cell development, and the role of pattern recognition receptors for bacteria and viruses. Among the genes that were strongly modulated in neonates with NEC, we observed a significant degree of similarity when compared with those reported in Crohn’s disease, a chronic inflammatory bowel disease.

**Conclusions:**

Gene expression profile analysis revealed a predominantly altered immune response in the intestine of NEC neonates. Moreover, comparative analysis between NEC and Crohn’s disease gene expression repertoires revealed a surprisingly high degree of similarity between these two conditions suggesting a new avenue for identifying NEC biomarkers.

**Electronic supplementary material:**

The online version of this article (doi:10.1186/s12920-016-0166-9) contains supplementary material, which is available to authorized users.

## Background

Necrotizing enterocolitis (NEC) is the most common life-threatening gastrointestinal disease of premature infants occurring in neonatal intensive care units [1, 2]. NEC is associated with severe intestinal inflammation, intestinal necrosis and high morbidity [3]. Survivors of NEC are at higher risk for developing short bowel syndrome, cholestatic liver disease as well as impaired growth and neurodevelopmental outcomes [4]. Several epidemiological risk factors have been proposed to play major roles in the pathogenesis of NEC, including preterm birth, enteral feeding and abnormal bacterial colonization [5, 6]. Only prematurity has been recognized in the literature as an established risk factor for NEC, although the exact mechanism has not yet been fully elucidated [1, 2].

The greatest challenge for neonatologists is to identify reliable early clinical signs and symptoms of NEC [1, 2]. While there are multiple NEC-like conditions with various presentations, the most common form of the disease, referred to as “classic NEC”, is an inflammatory intestinal condition in prematurely born infants [1–3]. However, the early clinical manifestations of NEC are relatively nonspecific and can be easily misinterpreted as other gastrointestinal problems [1, 2, 7]. Given its unpredictable onset, at diagnosis NEC is often already at an advanced stage due to the initially insidious and then fulgurating progression of the disease [8, 9]. One strategy to prevent or treat NEC would be to develop an early diagnostic tool allowing the identification of preterm infants either at risk of developing NEC or at the onset of symptoms to aid in the diagnostic dilemma. Several attempts have been made to identify biomarkers in preterm infants with NEC [10–12] or distinguish it from related pathologies [13] but the ideal biomarker remains to be identified [14].

Over recent years, the development of high-throughput sequencing of RNA transcripts (RNA-Seq) has become an emerging tool for transcriptional profiling of differentially expressed genes [15, 16]. The objective of this study was to take advantage of this approach to determine the complete gene expression profiles of ileal specimens resected from preterm infants diagnosed with NEC vs non-NEC conditions to identify pathways that could lead to more insight into the pathogenesis of NEC in premature infants. As NEC is a relatively uncommon disease for which surgical intervention does not necessarily result in an improved survival rate and as such is becoming more and more avoided [17], specimens from NEC patients are rare, most notably those with mRNA quality sufficient for being used in RNA-Seq studies. For these reasons and in conjunction with the fact that the aim of the study was to identify general molecular markers for NEC screening, we chose to combine all available ileal NEC specimens that fulfill the mRNA criteria for RNA-Seq for this study. This approach has been used in the past [13] although it is more and more accepted that NEC characteristics for 25-28 w vs 29-32 w preterm are not identical.

Our analysis revealed that multiple components of the immune response were strongly modulated in the small intestine of neonates with NEC. The data support the suggestion that the development of NEC is related to the immaturity of the intestinal mucosa in dealing with an altered microbiome [1, 2, 4, 18, 19]. Since a defect in the immune response is also a landmark of Crohn’s disease (CD) [20–22], a chronic inflammatory bowel disease that also preferentially affects the terminal small intestine, we investigated whether NEC shares common functional alterations with CD. To address this question, the RNA-Seq data generated herein for NEC were compared with available microarray data generated from ileal CD samples from 4 studies [23–26], leading to the identification of several common functional and canonical pathways including genes under evaluation for their usefulness as CD biomarkers that could be of interest for the non-invasive diagnosis of NEC.

## Methods

### Study population and informed consent

This multi-centre collaborative study recruited premature infants from neonatal intensive care units at the Centre Hospitalier Universitaire de Sherbrooke (Sherbrooke, QC, Canada), Erasmus MC-Sophia Children’s Hospital (Rotterdam, The Netherlands), Children’s Hospital of Eastern Ontario (Ottawa, ON, Canada) and Hôpital Pierre Zobda-Quitman (Fort-de-France, Martinique) between October 2008 and May 2013. Prior approval of the local Institutional Review Committees for the use of human material was obtained at each center. The overall project was approved by the Ethic Review Board on Human Health Research of the Centre Hospitalier Universitaire de Sherbrooke. Written informed consent from parents or guardians was obtained for each patient.

Premature infants having undergone bowel resection were eligible for the study. The diagnosis was confirmed by pathologists and clinical staging of NEC were based on the criteria of Bell et al. [8]. Freshly resected intestinal specimens taken from ileum were preserved in RNAlater (Ambion) before RNA extraction. Preterm patients who had undergone bowel resection for stage III acute NEC constituted our positive NEC cases and preterm patients who had undergone resection for diseases other than NEC made up the control (CTRL) group, as detailed in Table 1.

**Table 1:** Patient characteristics

Patient #	Sex	GA at birth (wk)	Birth weight (g)	GA at surgery (wk)	Diagnosis at surgery	Location
CTRL						
1	F	33 5/7	2001	33 6/7	Small intestinal perforation	ileum
2	F	26 6/7	905	29 4/7	Milk curd syndrome	Ileum, proximal
3	M	26 5/7	750	31	Meconium ileus	ileum
4	M	33 6/7	1675	34 1/7	Bowel obstruction	ileum
5	F	39	3635	39 2/7	Small intestinal atresia	ileum
6^a^	M	33 4/7	1779	33 4/7	Omphalocele	Ileum
NEC						
7	M	32	1500	40	NEC	Ileum
8	M	25 3/7	820	29	NEC	Ileum, proximal
9	F	26 2/7	690	36	NEC	Ileum
10	M	27 2/7	842	27 4/7	NEC	Ileum
11	F	24 6/7	600	26	NEC	Ileum, terminal
12	F	26 2/7	870	35 1/7	NEC	Ileum
13	M	25 4/7	830	27 1/7	NEC	Ileum
14	F	29 6/7	1160	30 6/7	NEC	Ileum, terminal
15	F	29 2/7	n/a	35 6/7	NEC	Ileum, terminal

*BW* birth weight, *F* female, *GA* gestational age, *M* male; ^a^ only used for qPCR

### Sample preparation and RNA sequencing

Total RNA from intestinal specimens was extracted using the RNeasy Lipid Tissue total RNA mini kit (Qiagen, Valencia, CA). Extracted RNA samples underwent quality control assessment using the Agilent Bioanalyzer (Agilent, Santa Clara, CA) and all RNA samples submitted for sequencing had an RNA Integrity Number >7. Poly-A library preparation and sequencing were performed at the McGill University and Génome Québec Innovation Centre (Montréal, QC, Canada) as per standard protocols. Briefly, ribosomal RNA from each RNA sample was removed using TruSeq Stranded Total RNA with Ribo-Zero for Human (Illumina, San Diego, CA), then first-strand cDNA was generated using random hexamer-primed reverse transcriptase, followed by second-strand cDNA synthesis using RNase H and DNA polymerase, and ligation of sequencing adapters using the TruSeq RNA Library Preparation Kit (Illumina, San Diego, CA). The prepared libraries were then sequenced using Illumina’s HiSeq 2000 to obtain 50-bp single-end reads using four lanes (4 samples per lane). Sequence data quality check was performed using FastQC (v1.0.0).[27] The RNA-Seq data were mapped to the hg19 reference genome using TopHat for Illumina (v1.5) using default options. Assembly of transcripts and estimation of their abundance (FPKM: fragments per kilobase of exon per million fragments mapped) were calculated using Cufflinks software (v0.0.6) [27]. We used the program Cuffdiff (v0.0.7) [27] to test for differential transcript expression between CTRL and NEC (*p* < 0.05).

### Functional pathway enrichment analysis

Ingenuity Pathway Analysis (IPA; Ingenuity Systems Inc., Redwood City, CA, USA) and ToppCluster [28] were used to identify functional pathway enrichment involved in NEC and CD. IPA generated a score for each predefined canonical pathway, which gave the likelihood that the set of genes in this pathway could be explained by chance alone. Canonical pathways with a score ≥2 have ≥99 % confidence that they are not generated by chance. ToppCluster generated *P* values (*P* < 0.05 with FDR correction) for human and mouse phenotypes associated with up-regulated or down-regulated genes in NEC and CD.

### RNA amplification and data validation by qPCR

Total RNA from 15 samples (fourteen used for RNA sequencing analysis plus one late additional sample added for reverse transcriptase-qPCR) was first amplified using the « TargetAmp™ 2-Round aRNA Amplification Kit 2.0 » (Epicentre Biotechnologies, Madison, WI) according to the manufacturer’s protocol. First-strand cDNA synthesis using Superscript II (Invitrogen) was performed on 1 μg total RNA using oligo (dT) _12–18_ as primer. All qPCR reactions were performed in duplicate using 25 ng of input template as previously described [29]. Amplification efficiencies ranged from 93 % to 104 % and the absence of primer-dimers was verified post-amplification by melting curve analysis. The genes investigated were beta-actin (ACTB), beta-2-microglobulin (B2M), chemokine (C-X-C motif) ligand 8 (CXCL8) and 10 (CXCL10), alpha-defensin 5 (DEFA5) and 6 (DEFA6), hemoglobin subunits (HBA2 and HBG2), lipocalin 2 (LCN2), regenerating islet-derived 3 alpha (REG3A), trefoil factor 1 (TFF1) and 3 (TFF3), Toll-like receptor 4 (TLR4) and 10 (TLR10). Primers (listed in Additional file 1) were generated using the primer formation software Primer3 (http://bioinfo.ut.ee/primer3). Differences in gene expression were evaluated by comparing reversed ∆Ct (r∆Ct = Ct_reference gene_–Ct_target gene_) of CTRL vs NEC samples using B2M as the validated reference gene [30] (same results were obtained using ACTB).

## Results

### RNA-Seq analysis and identification of differentially expressed genes (DEGs)

RNA-Seq analysis of intestinal samples generated 2231 × 10^6^ base pairs (bp) from NEC and 1589 × 10^6^ bp from CTRL. Mapping resulted in 44.63 × 10^6^ (±8.7 × 10^6^) reads in NEC and 31.79 × 10^6^ (± 0.12 × 10^6^) in CTRL. In total, 24346 genes were identified in both preterm intestinal samples. The data have been deposited in the National Center for Biotechnology Information’s Gene Expression Omnibus and are accessible through GEO Series accession number GSE64801.

We used the Illumina HiSeq2000 to investigate the gene expression profiles of the ileum of preterm infants with NEC vs without NEC (CTRL). In total, 804 DEGs (*p* < 0.05) were identified, 383 up-regulated and 421 down-regulated genes (See Additional file 2 for the gene list with fold changes).

### Functional pathways analysis in NEC

**Fig. 1:**
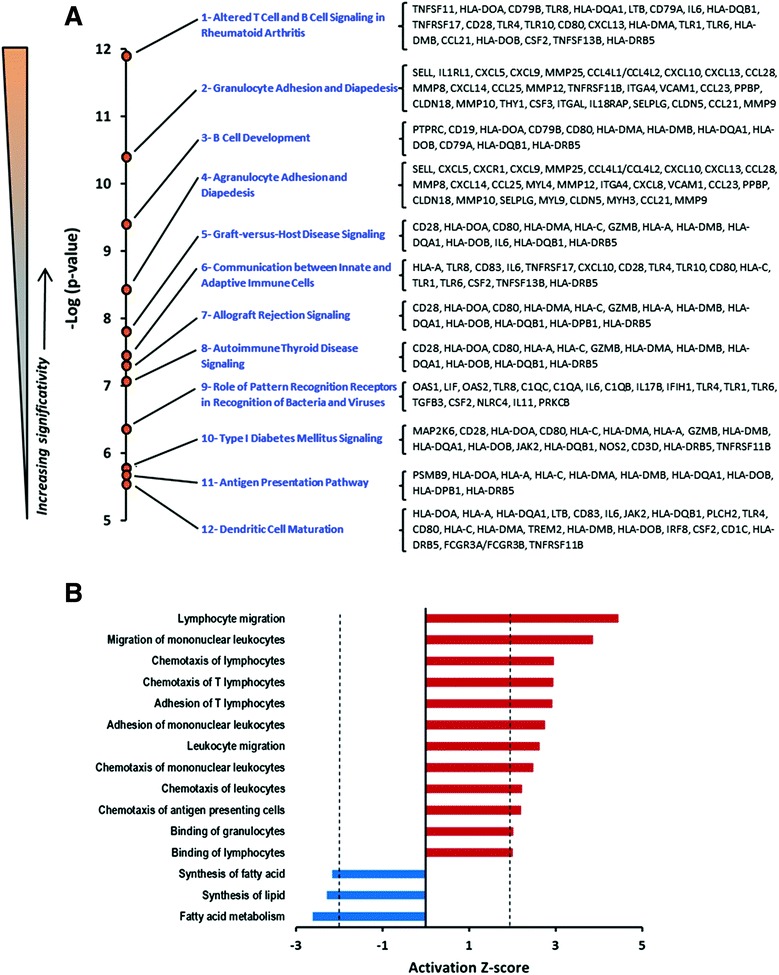
Most significant functional pathways identified in necrotizing enterocolitis. **a** The negative logarithm of *p*-values (Fisher’s test), calculated by IPA, for each of the top 12 most significant canonical pathways over-represented in ileal NEC samples compared to control non-NEC samples. ([-Log (0.05) =1.3]) and the corresponding lists of genes associated with each functional pathway. **b** Biological function enrichment analyses associated with NEC. Activation z-score calculated by IPA for biological function enrichment represents the level of activation (red) or suppression (blue) of a function

To identify functional and canonical pathways involved in the pathogenesis of NEC, the DEGs were submitted to IPA core analysis. The top twelve most significant canonical pathways modulated between NEC and CTRL and their associated genes are displayed in Fig. 1a (See Additional file 3 for the complete list of pathways and associated genes). Interestingly, most significant canonical pathways over-represented in the intestine of NEC neonates were associated with innate immune functions, such as altered T and B cell signaling, granulocyte adhesion and diapedesis, B cell development and the role of pattern recognition receptors for bacteria and viruses. In addition, ToppCluster analysis identified several biological functions as being altered in NEC (Fig. 1b) including up-regulation of lymphocyte and leukocyte migration, T lymphocyte and antigen presenting cells chemotaxis, adhesion of T lymphocytes, leukocytes and granulocytes and down-regulation of functions related to lipid metabolism, establishing a signature of biological functions associated with NEC.

### Validation of the gene expression profile of NEC

**Fig. 2:**
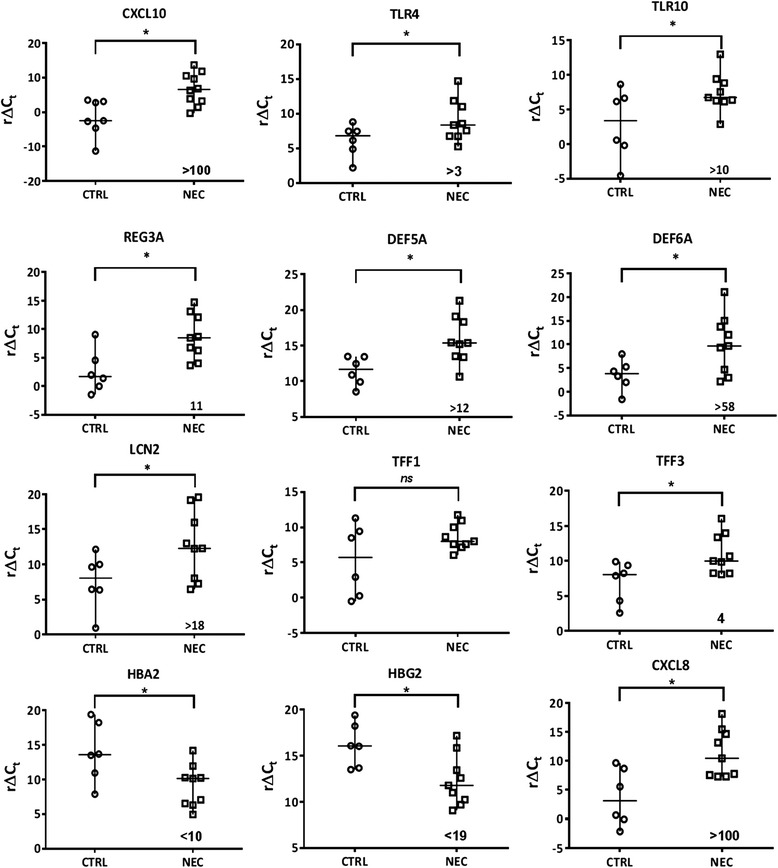
Differential expression of innate immune inflammatory response genes in human necrotizing enterocolitis. Real-time qPCR analysis of transcript levels of selected target genes related to intestinal innate immunity. Ct values of selected genes were normalized using B2M as reference gene and data are expressed as r∆Ct values (reverse ∆Ct: Ct_reference gene_-Ct_gene of interest_) in order to display direct variation in NEC vs non-NEC controls (CTRL). Horizontal line represents the median value of r∆Ct values for CTRL and NEC samples. *: *p* <0.05 between CTRL and NEC samples. Numbers indicated represent the fold variation between NEC and CTRL

To further validate the gene expression profiles identified by RNA-Seq analyses, we used qPCR to test representative DEGs in NEC samples among those known to be involved in the inflammatory processes, innate immunity and antimicrobial responses: CXCL10, TLR4, TLR10, DEFA5, DEFA6, REG3A, LCN2, TFF3, HBA2 and HBG2. Transcript levels of TFF1 and CXCL8 were also determined although these 2 genes were not identified as DEGs by RNASeq. As shown in Fig. 2, qPCR analyses confirmed the up-regulation of CXCL10, TLR4, TLR10, DEFA5, REG3A, LCN2 and TFF3 and down-regulation of HBA2 and HBG2 expression in NEC. As expected, TFF1 was not modulated but CXCL8 levels were found to be significantly up-regulated in NEC samples (Fig. 2). The lack of detection of CXCL8 by RNA-Seq in NEC at statistically significant levels can be explained by the high variability in its expression in both NEC and CTRL neonatal intestines as observed by qPCR (Fig. 2) and the fact that shorter transcripts such as CXCL8 are less efficiently detected by the short read procedure used in RNA-Seq [31]. Taken together, these gene expression profiling results suggest that specific alterations in the intestinal innate immune response could contribute to the pathogenesis of NEC.

### Comparison between NEC and CD expression profiles

**Fig. 3:**
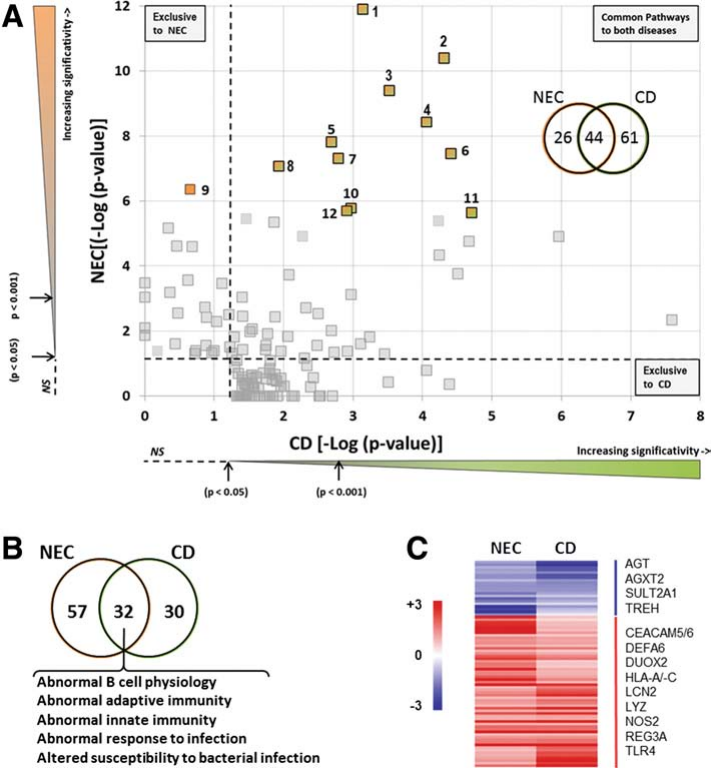
Comparative analysis of functional enriched pathways between necrotizing enterocolitis and Crohn’s disease. **a** The negative logarithm of *p*-values (Fisher’s test), calculated by IPA, for each of the functional categories over-represented in NEC samples was plotted against those modulated in Crohn’s disease according to published data [23–25]. Canonical pathways represented by colored squares indicating the top 12 functional pathways identified in NEC are listed in Fig. 1. As shown, 11 of them are shared between NEC and CD. *Insert*: Venn diagram showing the 131 canonical pathways between NEC and CD. Of the 70 pathways found in NEC, 44 were also found in CD. Thresholds (dotted lines) denote the limit of statistical significance (*p* = 0.05 [-Log (0.05) =1.3]). **b** Venn diagram showing ToppCluster enrichment analysis associated with NEC patients and adult CD using phenotype terms. **c** Heatmap of some common genes found in the intersection of the ToppCluster enrichment analysis between NEC and adult CD as detailed in Additional file 6: Table S6

Considering that dysregulation in the innate immune response is a landmark of CD [20–22], one of the most common inflammatory bowel diseases that also predominantly affects the ileocecal region, we undertook a systematic comparison of the DEGs observed herein in the ileum of NEC with those reported in the ileum of adult patients with active CD [23–25] followed by a comparative functional analysis with IPA software. As used previously to compare gene clustering under two conditions [29, 32, 33], we plotted the negative logarithm of *p*-values calculated by IPA for each of the functional categories found in NEC against the negative logarithm of *p*-values of the corresponding categories found in CD in order to identify the relationship between individual functions in the two diseases (Fig. 3a). Overall, we noted that more than 60 % of the significant pathways identified in NEC were also identified in CD (Fig. 3a, insert). Interestingly, 11 of the 12 most significant common canonical pathways identified in NEC (Fig. 1a) were found among those also significantly altered in CD (Fig. 3a; see Additional file 4 for the list of the 103 significant pathways and corresponding DEGs in CD and Additional file 5 for the list of the 44 common pathways) including T and B cell signaling, diapedesis and autoimmune response. Gene set enrichment analysis using ToppCluster [28] with DEGs identified for both NEC and CD, confirmed the closeness of the two diseases by demonstrating several common gene families related to immunity and infection (Fig. 3b). It is noteworthy that at the individual gene level, 175 (21.8 % of the total) of the DEGs identified in NEC also appear to be significantly altered in human ileal CD [23–25] (see Additional file 6 for a complete list of common DEGs between NEC and CD). Also included are genes involved in antimicrobial activity such as DEFA6, DUOX2, LCN2 and LYZ as well as other important genes involved in mucosal immunity (Fig. 3c).

**Fig. 4:**
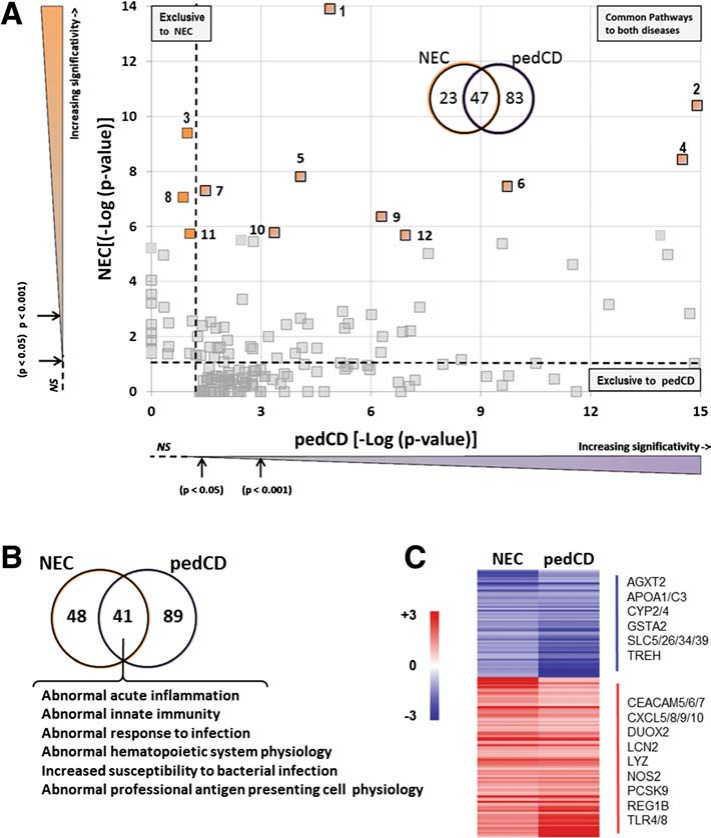
Comparative analysis of functional enriched pathways between necrotizing enterocolitis and pediatric Crohn’s disease. **a** The negative logarithm of *p*-values (Fisher’s test), calculated by IPA, for each of the functional categories over-represented in NEC samples was plotted against those modulated in pedCD according to published data. [26] Canonical pathways represented by colored squares indicate the top 12 functional pathways identified in NEC as listed in Fig. 1. As shown, 9 of them are shared between NEC and CD. *Insert*: Venn diagram showing the 153 canonical pathways identified in NEC and pedCD. Of the 70 pathways found in NEC, 47 were also found in pedCD. Thresholds (dotted lines) denote the limit of statistical significance (*p* = 0.05 [-Log (0.05) =1.3]). **b** Venn diagram showing ToppCluster enrichment analysis associated with NEC patients and pedCD using phenotype terms. **c** Heatmap of some common genes found in the intersection of the ToppCluster enrichment analysis between NEC and pedCD as detailed in Additional file 8: Table S8

Recently, Haberman et al. [26] have reported specific gene expression profiles in pediatric ileal CD (pedCD) patients. By conducting comparative functional analyses by IPA and ToppCluster using NEC vs pedCD gene expression profiles, we determined that NEC also shares a large number of functional canonical pathways (Fig. 4a; see Additional file 7 for the list of the 130 significant pathways and corresponding DEGs in pedCD) and specific disease phenotypes (Fig. 4b) with pedCD (67 and 46 %, respectively). At the individual gene level, 197 (25 % of the total) of the DEGs identified in NEC also appear to be modulated in pedCD [26] (see Additional file 8 for a complete list of common DEGs) such as CXCL10, DUOX2, LCN2 and LYZ (Fig. 4c).

However, our analysis also revealed individual genes that were exclusively modulated in the small intestine of neonates with NEC but not in CD or pedCD. A few of these genes are TLR10, DEFA5, TFF3, HBA2 and HBG2 (Fig. 2). Even if these genes were found in functional canonical pathways common to CD, their gene expression profiles were specifically altered in NEC.

### Distinctive upstream regulators in NEC

**Fig. 5:**
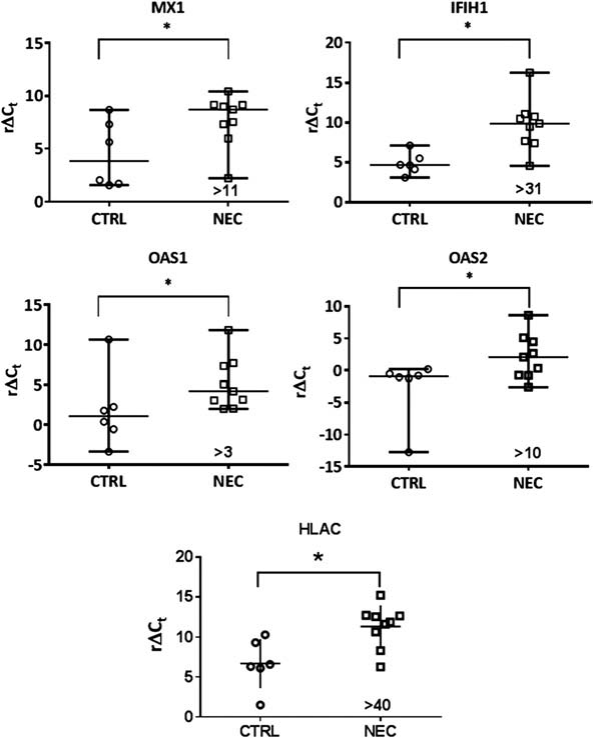
Differential expression of antiviral response genes in human necrotizing enterocolitis. Real-time qPCR analysis of transcript levels of selected target genes related to the antiviral response. Ct values of selected genes were normalized using B2M as reference gene and data are expressed as r∆Ct values (reverse ∆Ct: Ct_reference gene_-Ct_gene of interest_) in order to display direct variation in NEC vs non-NEC controls (CTRL). Horizontal line represents the median value of r∆Ct values for CTRL and NEC samples. *: *p* <0.05 between CTRL and NEC samples. Numbers indicated represent the fold variation between NEC and CTRL

To further identify biological processes specifically involved in the pathogenesis of NEC, we compared IPA upstream regulator analyses between NEC and CD, including pedCD, and found that six upstream transcriptional regulators were exclusively altered in NEC (Table 2). Interestingly, these upstream regulators were involved in antiviral and antimicrobial host defense. We validated the gene expression profiles of some representative genes in NEC samples known to be involved in the antiviral or antimicrobial responses: IFIH1, MX1, OAS1, OAS2 and HLAC (Fig. 5). Taken together, these distinctive upstream regulator analyses suggest that the antiviral or antimicrobial response has been triggered in the intestinal mucosa of NEC neonates and could specifically participate in the pathogenesis of NEC.

**Table 2:** Exclusive upstream regulators in human necrotizing enterocolitis

Upstream Regulator	Molecule Type	Predicted Activation State	Activation z-score	Target molecules in dataset
EBI3	Cytokine	Activated	2.646	CD80, HLA-A, HLA-C, HLA-DMA, HLA-DMB, HLA-OB, HLA-DQA1
KDM5B	Transcription regulator	Activated	2.343	CAV1, GAL, GCA, MCAM, MT1H, MT1X, PTPLA, REEP1, TUBB2A
JAK1	Kinase	Activated	2	HLA-A, HLA-C, IFIT2, MX1
TICAM1	Other	Activated	2	CXCL10, IFIT1, IFIT2, IL8
SOCS3	Phosphatase	Inhibited	−2.433	CXCL10, IFIT1, IFIT2, IL6, MX1, OAS1, OAS2
SOCS1	Other	Inhibited	−2.586	CXCL10, IFIH1, IFIT1, IFIT2, MX1, OAS1, OAS2

## Discussion

To further investigate the functional processes underlying NEC pathogenesis at the molecular level, we have used high-throughput mRNA sequencing analysis combined with enrichment analysis tools on ileal samples obtained from premature neonates affected with NEC vs CTRL to fully characterize the repertoire of NEC-related gene expression. Our results showed that the most significant biological pathways altered in NEC are those encoding immune functions such as T and B cell signaling, B cell development and dendritic cell maturation, diapedesis and role of pattern recognition receptors for bacteria and viruses. A previous study using microarrays to investigate gene expression profiles in a limited number of NEC samples of small and large intestines (*n* = 5) also identified the immune response among the significantly altered biological processes in NEC but the lack of individual gene listing [13] prevented any direct comparison. Nevertheless, herein, modulation in the expression of pro-inflammatory cytokines, Toll-like receptors, antimicrobial molecules and hemoglobin subunits were noted at the individual gene level and confirmed by qPCR.

Increased expression of CXCL8/IL8 in NEC is in agreement with previous studies [19] which report an excessive inflammatory response in the immature intestine. Like CXCL8/IL8, up-regulation of CXCL10 was also noted in the intestine of preterm neonates [34]. Interestingly, elevated circulating levels of both of these cytokines were recently noted in preterm infants diagnosed with NEC [35]. Up-regulation of TLR4 and TLR10 in NEC neonates as compared to non-NEC preterm neonates is consistent with the key role played by these bacteria sensing molecules in infectious diseases [20]. Indeed, several lines of evidence have demonstrated the central importance of the bacterial lipopolysaccharide receptor TLR4 in many aspects of NEC pathogenesis in the context of an immature innate inflammatory response leading to apoptosis, autophagy, proliferation and cell differentiation [36, 37]. Modulation of TLR10 expression has not been described previously in the intestine of NEC patients but has recently been reported to act as an anti-inflammatory pattern-recognition receptor [38]. TLR10 is one of the few Toll-like receptors without known ligand specificity but recent work in intestinal cells suggests that it could mediate the inflammatory response to Listeria monocytogenes [39] and an association of the TLR10 gene with CD susceptibility has been reported [40].

Up-regulation of intestinal antimicrobial peptide expression in NEC is another indication supporting immaturity in the innate immune response in this disease. Among these genes were the two main α-defensins expressed in the human intestine: DEF5A and DEF6A. Both defensins are produced by Paneth cells along with a panel of other antimicrobial peptides and proteins that include REG3A, LYZ and PLA2G2A [41], also found herein to be up-regulated in NEC samples by RNA-Seq. The microbiocidal activity of α-defensins against Gram-positive and Gram-negative bacteria, certain fungi, spirochetes, protozoa and enveloped viruses has been well demonstrated for DEFA5 [41, 42]. DEFA6 appears to be able to kill specific microbes under certain conditions [43] in addition to its ability to form nanonets to entrap pathogenic bacteria [44]. Antimicrobial activity has also been reported for REG3A [45]. These results are consistent with the fact that Paneth cells have been suggested to be involved in NEC pathogenesis [46, 47]. However, in contrast to CD [41, 42], no susceptibility gene has yet been identified in NEC. In fact, Paneth cell abundance in preterm infants with NEC in comparison to preterm controls was found to be comparable [47] or even increased [48]. Interestingly, Paneth cell hyperplasia and metaplasia was noted in infants recovering from NEC while Paneth cell products obtained from NEC patients displayed strong antimicrobial activity, suggesting that Paneth cells are at least partially functional in this disease [47]. A recent interesting hypothesis suggests that other Paneth cell products such as the pro-inflammatory cytokine TNFα and IL-17 could trigger the inflammatory process in NEC [46]. While neither TNFα nor IL-17 was found to be up-regulated in the intestine of patients affected by NEC in the present study, the possibility that an acute inflammatory response could be initiated by Paneth cells cannot be ruled out.

The increase of LCN2 encoding neutrophil gelatinase-associated lipocalin/lipocalin-2 in NEC samples could be of interest for diagnostic purposes. Indeed, this antimicrobial molecule has been reported to be up-regulated in intestinal cells in response to a variety of pro-inflammatory stimuli [49] and can serve as an efficient blood and fecal biomarker for monitoring inflammatory bowel diseases in the adult [50, 51]. Incidentally, TFF3, also identified among the up-regulated genes expressed in NEC in this study, is one of the recently identified gut-associated serum markers for the diagnosis of NEC [52].

Another interesting finding from this study is the significant decrease observed in the expression of all hemoglobin subunits in the intestine of NEC cases including α, β, δ and γ hemoglobin subunits as well as AHSP, the α-hemoglobin chaperone, which was confirmed by qPCR (AHSP; not shown). Although not yet described in the intestine, non-erythroid hemoglobin expression has been reported in various cell types where it exerts antimicrobial activity and plays a role in oxidative and nitrosative stresses [53]. Considering the hematologic abnormalities associated with the development of NEC [1, 2], future investigation of hemoglobin is needed in light of a recent study that reported a toxic effect of α-hemoglobin on colonic epithelial cells [54].

Finally, we also identified six cascades of upstream transcriptional regulators that were exclusively modulated in NEC. Among these regulators, we observed that SOCS1 and SOCS3 were predicted to be inhibited in NEC. SOCS proteins play major roles in inflammatory diseases and infection by suppressing cytokine signaling [55]. It has been previously reported [56] that SOCS1 and SOCS3 inhibit the expression of OAS1 and MX1 [57, 58], two key antiviral effectors. The up-regulation of antiviral genes MX1, OAS1 and OAS2 that we observed in NEC confirmed the inhibited state of SOCS1 and SOCS3, and suggests that an antiviral response could have been triggered and play a role in the pathogenesis of NEC.

Taken together, these results suggest that the mucosa of the intestine of neonates affected by NEC has been exposed to a disproportionate inflammatory response likely due to an immature innate immune response concurrent with an altered microbiota composition and overgrowth of specific microorganisms. It is noteworthy that many of the genes identified to be modulated in neonates with NEC have also been proposed to be involved in chronic inflammatory bowel disease, even though NEC is considered to be an acute condition. To further investigate this question, we compared our RNA-Seq results on NEC with available microarray data from adult [23–25] and pediatric [26] CD. Our results confirm that a large proportion of the significant functional pathways and phenotypes are common between NEC and CD. More importantly, 22 and 25 % of the DEGs identified in NEC also appear to be modulated in CD [23–25] and pedCD [26], respectively. As mentioned above, these DEGs include specific cytokines and TLRs, antimicrobial peptides such as α-defensins and REGs, as well as the multifunctional proteins LCN2 and TFF3 that are all up-regulated. Recently, it has been reported that a specific ileal gene expression profile in pediatric CD is associated with a depletion of a specific microbial community [26]. The possibility that the NEC-associated ileal gene expression pattern could be linked to a specific microbial signature in NEC preterms could be an interesting avenue and needs to be investigated. In a context where there is still a crucial need for the characterization of reliable predictive markers for NEC development [14], our observations suggest that the some of the biomarkers identified to be of good diagnostic value for CD could be useful in the pediatric intensive care unit as non-invasive markers to predict NEC development, such as LCN2 [50, 51] for instance. This approach is not without precedent, as recently demonstrated for calprotectin [59].

## Conclusions

In conclusion, this study has led to the identification of several DEGs in intestinal samples of premature infants affected with NEC that could be of clinical interest as potential biomarkers for the prediction of the disease and its diagnosis. Furthermore, considering that a significant proportion of the DEGs are common with those identified in CD, a widespread intestinal condition for which the biomarker pipeline is much more advanced, our observations suggest that the evaluation of some of the characterized CD biomarkers could also be useful for a non-invasive diagnosis of NEC.

## Additional files

Additional file 1: **Primers used for qPCR in this study.** (XLS 48 kb)

Additional file 2: **List of differentially expressed genes in NEC.** (XLS 137 kb)

Additional file 3: **List of canonical pathways modulated in NEC.** (XLS 46 kb)

Additional file 4: **List of canonical pathways modulated in CD.** (XLS 66 kb)

Additional file 5: **List of canonical pathways modulated in NEC and CD.** (XLS 50 kb)

Additional file 6: **List of differentially expressed genes in NEC and CD.** (XLS 68 kb)

Additional file 7: **List of canonical pathways modulated in pedCD.** (XLS 68 kb)

Additional file 8: **List of differentially expressed genes in NEC and pedCD.** (XLS 68 kb)

## Abbreviations

CD: crohn’s disease
CTRL: control
DEGs: differentially expressed genes
IPA: Ingenuity pathway analysis
NEC: necrotizing enterocolitis
pedCD: pediatric CD
qPCR: quantitative polymerase chain reaction
RNA-Seq: high-throughput sequencing of RNA transcript
